# Cleft Laterality Dental Arch Relationship Outcomes for Children with Unilateral Cleft Lip and Palate in New Zealand

**DOI:** 10.1177/10556656241234599

**Published:** 2024-02-27

**Authors:** Peter V. Fowler, H. Keall, D. Kennedy, D. Healey, J.M.D. Thompson

**Affiliations:** 1 Department of Oral Sciences, University of Otago Dental School, Dunedin, New Zealand; 2 Formerly Hospital Dental Department, 1400 Middlemore Hospital, Auckland, New Zealand; 3 Hospital Dental Department, 1400 Middlemore Hospital, Auckland, New Zealand; 4 Formerly School of Dentistry, University of Queensland, Brisbane, Australia; 5 Department of Obstetrics and Gynaecology, Faculty of Health Sciences, University of Auckland, Auckland, New Zealand; 6 Department of Paediatrics, Child and Youth Health, University of Auckland, Auckland, New Zealand

**Keywords:** cleft laterality, UCLP dental arch, outcomes

## Abstract

**Objectives:**

To investigate cleft laterality dental arch relationship outcomes of children with non-syndromic complete unilateral cleft lip and palate (UCLP) in New Zealand.

**Design:**

A retrospective nationwide study.

**Settings:**

Virtual 3D orthodontic study models collected prior to undertaking secondary alveolar bone grafting.

**Participants:**

A total of 104 patients with UCLP (L = 80: R = 24).

**Outcome measures:**

Four calibrated assessors used the GOSLON Yardstick and 100 mm Visual Analogue Scale (VAS) to score the randomised models on 2 separate assessment sessions. Weighted Kappa were used to determine the intra/inter-rater reliability for the GOSLON and correlations for the VAS.

**Results:**

Intra-rater reliability ranged from 0.57-0.88 (GOSLON) and 0.45-0.93 (VAS). Inter-rater reliability ranged from 0.62-0.86 (GOSLON) and 0.64-0.93 (VAS).

GOSLON scores for the left UCLP were 31.2% for good/very good; 26.3% for fair; 42.5% for poor/very poor while the right UCLP scored 8.3% for good/very good; 37.5% for fair; 54.2% for poor/very poor. The mean VAS for left and right UCLP were 53.4 (sd 22.5) and 44.6 (sd 17.1) respectively. Neither the GOSLON nor VAS differences reached statistical significance (both *P* = .08).

**Conclusions:**

From a clinical perspective right UCLP had worse dental arch relationship outcomes, however, these differences failed to reach statistical significance. Further studies using larger sample sizes are required to determine if cleft laterality is an important consideration when investigating UCLP dental arch outcomes.

## Introduction

Cleft laterality incidence with unilateral orofacial clefts is relatively consistent where left-sided cleft occurs approximately 2:1 in relation to those on the right. This occurs despite differences in incidence of orofacial cleft (OFC) among different populations and geographic locations.^1^ This non-random occurrence could be considered an example of directional asymmetry.^2^ Similar patterns of asymmetry seen in paired structures of the face include a propensity for left sided agenesis of hypodontia irrespective of cleft sidedness^3^ and right sided hemifacial microsomia.^4^ This suggests that developmental differences may exist between the paired facial structures, where one side has a greater liability for a defect in morphogenesis to occur.^5^

Although the aetiology of OFC has been under extensive investigation with multiple genetic and environmental factors implicated, investigations into the mechanism of orofacial cleft laterality are limited and their findings inconclusive. Anatomical considerations include asymmetric blood supply due to the right internal carotid, which leaves the aortic arch closer to the heart, providing higher blood pressure^6^ and/or slower facial artery development on the left side^7^ which may leave the left side more susceptible to hypoxia during early embryogenesis.^8^ Environmental considerations are inconclusive but include maternal smoking which has been reported to be associated with left sided cleft lip (CL) predominance,^9^ whereas right sided cleft lip and palate (CLP) predominance has been reported with maternal smoking.^10^ Moreso, the correlation of maternal smoking may be a sex-specific environmental determinant of laterality, as the right sided CLP predominance was reported only for girls.^10^ Although many genetic studies have been undertaken to better understand the role of genetics in OFC, limited investigations have been undertaken determine individual cleft subtypes including laterality. Recent genetic investigations using genome-wide modifying analysis within a multi-ethnic case control study have reported potential involvement Fat4 gene on chromosome 4q28 as a modifier of CL laterality but not CLP.^11^ Fat4 has been reported to influence the mesenchyme of the medial nasal processes and primary palate. The study's findings are suggestive that potential genetic modifiers of laterality in CL may be distinct from those in CLP.^11^

To date, few studies have distinguished between left and right sided clefts when investigating outcomes. Previous outcome studies have often grouped left and right sided patients with cleft together as a homogenous group. However, a recent study reported cleft laterality anthropometric measurement differences for patients aged between 3-6 months of age presenting for primary surgical repair.^12^ Those patients with right sided clefts had greater lateral lip hypoplasia and greater deficiency in lateral lip vertical height and vermillion height when compared to left sided clefts. When outcome studies have reported on cleft laterality outcomes some have suggested there are differences and others not. Studies reporting poorer outcomes for right sided clefts compared to left sided cleft include facial appearance, brain structure, academic performance, psychosocial wellbeing, additional malformations and post treatment occlusion.^13-17^ Whereas other outcome studies have reported no difference associated with cleft laterality,^18-21^ or that left sided cleft had poorer outcomes.^22^

Currently there is a lack of studies reporting on cleft laterality outcomes using dental arch relationships. The GOSLON yardstick is considered as the gold standard in assessing dental arch relationship outcomes for patients with unilateral cleft lip and palate (UCLP) and has been used in major inter-centre outcome studies.^23^ The GOSLON Yardstick categorises five possible outcomes (very good to very poor) that represent the full range of possible anterior-posterior dental arch relationship outcomes for patients with UCLP in the early permanent or late mixed dentition.

Previously reported studies assessing dental arch relationships of children with UCLP in New Zealand (NZ) have reported poor GOSLON Yardstick outcomes.^24,25^ These outcomes were similar to those reported by Clinical Standards Advisor Group (CSAG) audit in the United Kingdom and those centres who reported the poorest scores from the Americleft and Eurocleft studies.^26-28^

The incidence of OFC in NZ is 1.79 per 1000 live births^29^ and cleft care is provided within 5 government funded cleft centres. The primary surgery protocols at these centres are similar with lip repairs undertaken between 3-6 months and palatal repair around 12 months. The 12 surgeons undertaking these repairs had new-born with cleft caseloads ranging from approximately 2-18 per year.^30^

Like many other studies reporting dental arch relationship outcomes for UCLP, the NZ studies assessed both left and right sided UCLP as a homogeneous group. Given the potential shortcomings of this approach and considering recent interest in cleft laterality outcomes, the aims of this study were to investigate cleft laterality dental arch relationship outcomes of children with non-syndromic complete UCLP in NZ. We hypothesised that there would be similar scoring between the left and right sided UCLP using either the GOSLON Yardstick or Visual Analogue Scale (VAS) assessments.

## Methods and Materials

This was a retrospective nationwide study used virtual 3D orthodontic study models from patients with non-syndromic complete UCLP born in NZ from 1st Jan 2000 onwards and where data collection was completed in 2017. The original orthodontic plaster models were collected prior to undertaking secondary alveolar bone grafting and any preparatory orthodontics. All models available were collected from patients with confirmed diagnosis of non-syndromic complete UCLP from the 5 cleft centres. For ease of scoring and blinding of the assessors, all the plaster models were scanned using a Maestro 3D Dental Scanner-MDS400 and provided with standardised virtual bases.

The virtual models were assessed by four calibrated assessors (XX, XY, ZZ, ZX) using 3D Maestro Ortho Studio 2.8 viewer software (AGE Solutions, Pontederia -Pisa, Italy). The models were assessed using the categorical GOSLON Yardstick scoring ranging from very good, good, fair, poor, very poor. In addition, the models scored using a 100 mm VAS where 0 mm represented the worst possible dental arch relationship and 100 mm the best possible dental arch relationship.^31^ The continuous nature of the VAS provided potential for greater statistical power and sensitivity of outcome assessment. Prior to scoring, the trained assessors were re-familiarized with the GOSLON Yardstick guidelines and categorical /VAS definitions and undertook calibration using virtual models not included in the study. Prior familiarisation of the 3D digital model viewing software on the computer was also undertaken. The origin of the 3D models was blinded to the assessors and the 3D models were randomised between 2 separate assessments. All the assessors, who were experienced orthodontists involved in cleft care, undertook the scoring in the same location with no conferring and had previously undertaken GOSLON Yardstick scoring accreditation.

The assessors were able to use a master set of GOSLON Yardstick reference scoring models, representing each of the five scoring categories as well as written categorical guidelines during the assessment. The scoring of the VAS was determined by similar assessment concepts as the GOSLON Yardstick and weighted Kappa were used to determine the intra/inter-rater reliability for the GOSLON Yardstick and correlations for the VAS. The consensus model score of the first rating session was used to establish the Yardstick rating while the VAS scores were averaged across the 4 scorers to provide a final mean score. All scoring results were entered into a spreadsheet (Microsoft Excel, Redmond, Washington) and Statistical Analysis Software (version 9.3; SAS Institute, Cary, North Carolina) was used for analysis. Chi-square tests were used to test for GOSLON Yardstick cleft laterality differences and a Student t test was used to test VAS score cleft laterality differences. Ethics approval for the wider cleft outcomes study was granted by the University of XX Human Participants Ethics Committee (012601).

## Results

A total of 104 models from patients with non-syndromic complete UCLP (L = 80:R = 24) were included in this study. It was estimated that the sample represented approximately 65% of all UCLPs with the shortfall relating to patients with syndromic and/or incomplete UCLP and those who had incomplete/absent dental models. The mean age of the patients at the time of model collection was 9.6 years (SD ± 1.6 years). Males represented 65% of the sample with the sample ethnicity mix reflecting that of the NZ general population (European 64%, Māori 16%, Pacific Island 9%, Other 11%).

Intra- and inter-rater reliability of the 100 mm VAS and the GOSLON Yardstick assessments are listed in Tables 1 and 2.

**Table 1.** Intra-rater Reliability.

	GOSLON Yardstick	VAS
Rater	Weighted Kappa	Correlations
1	0.88	0.89
2	0.88	0.82
3	0.65	0.89
4	0.57	0.64

Abbreviation: VAS, visual analogue scale.

**Table 2.** Inter-rater Reliability.

	GOSLON Yardstick (Weighted Kappa)			
Rater	1	2	3	4
1		0.86	0.68	0.69
2			0.72	0.72
3				0.62
4				
	VAS (Correlations)			
Rater	1	2	3	4
1		0.86	0.81	0.75
2			0.80	0.67
3				0.70
4				

Abbreviation: VAS, visual analogue scale.

Although right sided UCLP scored more poorly that the left sided UCLP, there were no statistical differences detected for cleft laterality outcomes for either the GOSLON Yardstick (Table 3) or the VAS assessments (Table 4) (both *P* = .08).

**Table 3.** GOSLON Yardstick Ranking Distribution of 3D Digital Models by Cleft Laterality.

GOSLON Yardstick	Left UCLP % (n = 80)	Right UCLP % (n = 24)
Very good / Good	31.2	8.3
Fair	26.3	37.5
Poor/Very Poor	42.5	54.2

**Table 4.** Visual Analog Scale Assessment of 3D Digital Models by Cleft Laterality.

VAS	Left UCLP (n = 80)	Right UCLP (n = 24)
Mean score (mm) ± SD	53.4 ± 22.5	44.6 ± 17.1

(Lower score = poorer outcome).

## Discussion

This study was undertaken to investigate cleft laterality outcomes assessing dental arch relationships. Intra and inter-rater reliability ranged from moderate to very good for the GOSLON Yardstick, while correlations for the VAS ranged from 0.45-0.93.

From a clinical perspective right UCLP had worse dental arch relationship outcomes with a higher percentage ranked in Poor/Very Poor category and lower percentage ranked in Very good/ Good category using the GOSLON Yardstick compared to left UCLP. It is likely that those patients who are ranked within the Poor/Very Poor category are likely require orthognathic surgery once facial growth has ceased. Orthognathic surgery is undertaken to address the skeletal discrepancy related to midface retrusion that are unlikely to be corrected by orthodontics alone.^32^ The right UCLP also had a worse (lower) mean VAS scorings compared to left UCLP, although large standard deviations and a lack of comparative data limit the clinical application of such a measure in this study. However, these laterality differences failed to reach statistical significance.

It is interesting to note that Staudt et al. reported better final dental arch relationship outcomes for left sided UCLP in a sample of 56 patients with UCLP following orthodontic and/or orthognathic treatment. These improvements were reported for the anterior and anterior-buccal arch segments, but the sample only included 10 right sided UCLP.^15^ When Chong et al. reported on cleft laterality unrepaired anthropometric measurement differences they also commented that some surgeons experience more difficulties with repair of right sided UCLP. They report that this may be due to actual anthropometric differences, the handedness of the surgeon and/or less familiarity with right sided UCLP repairs.^12^ However, to date, it remains unclear these difficulties lead to differences in cleft laterality outcomes.

This study's limitations in part reflects the difficulty in assessing cleft laterality outcomes for UCLP due to relatively small number of right UCLP compared to left UCLP and the risk of insufficient power to reach statistical significance. Although the collection of more right sided UCLP over a further extended time period is possible, it would also introduce confounders as the surgeons and surgical protocols have since changed in some cleft centres. Further studies using larger sample sizes are required to determine if cleft laterality is an important consideration when investigating UCLP dental arch outcomes. Preferably the data collection would be undertaken prospectively and where standardised collection of potential confounding variables can also be undertaken.

In addition, the intra/inter rater reliability, although ranged from moderate to very good for the GOSLON Yardstick was lower than other studies^27,28^ and may have reduced the ability to detect true differences or associations. The use of another outcome measures, such as the Modified Hubbart Bodenham Index to assess the occlusal relationships of the dental models could be considered which has been reported to be more objective than the GOSLON Yardstick.^23^

## Conclusion

The influence of cleft laterality on dental arch outcomes remains unclear and should be investigated further, either through individual pooled data analysis and/or using greater sample sizes of right sided UCLP.
